# Magnitude of Treatment Abandonment in Childhood Cancer

**DOI:** 10.1371/journal.pone.0135230

**Published:** 2015-09-30

**Authors:** Paola Friedrich, Catherine G. Lam, Elena Itriago, Rafael Perez, Raul C. Ribeiro, Ramandeep S. Arora

**Affiliations:** 1 Department of Pediatric Oncology, Dana-Farber/Boston Children’s Cancer and Blood Disorders Center, Boston, Massachusetts, United States of America; 2 Department of Oncology, St. Jude Children’s Research Hospital, Memphis, Tennessee, United States of America; 3 International Outreach Program, St. Jude Children’s Research Hospital, Memphis, Tennessee, United States of America; 4 Villa Victoria Center for the Arts, Boston, Massachusetts, United States of America; 5 Department of Medical Oncology, Max Healthcare, New Delhi, India; University of New Mexico Cancer Center, UNITED STATES

## Abstract

**Background:**

Treatment abandonment (TxA) is recognized as a leading cause of treatment failure for children with cancer in low-and-middle-income countries (LMC). However, its global frequency and burden have remained elusive due to lack of global data. This study aimed to obtain an estimate using survey and population data.

**Methods:**

Childhood cancer clinicians (medical oncologists, surgeons, and radiation therapists), nurses, social workers, and psychologists involved in care of children with cancer were approached through an online survey February-May 2012. Incidence and population data were obtained from public sources. Descriptive, univariable, and multivariable analyses were conducted.

**Results:**

602 responses from 101 countries were obtained from physicians (84%), practicing pediatric hematology/oncology (83%) in general or children’s hospitals (79%). Results suggested, 23,854 (15%) of 155,088 children <15 years old newly diagnosed with cancer annually in the countries analyzed, abandon therapy. Importantly, 83% of new childhood cancer cases and 99% of TxA were attributable to LMC. The annual number of cases of TxA expected in LMC worldwide (26,166) was nearly equivalent to the annual number of cancer cases in children <15 years expected in HIC (26,368). Approximately two thirds of LMC had median TxA≥6%, but TxA ≥6% was reported in high- (9%), upper-middle- (41%), lower-middle- (80%), and low-income countries (90%, p<0.001). Most LMC centers reporting TxA>6% were outside the capital. Lower national income category, higher reliance on out-of-pocket payments, and high prevalence of economic hardship at the center were independent contextual predictors for TxA ≥6% (p<0.001). Global survival data available for more developed and less developed regions suggests TxA may account for at least a third of the survival gap between HIC and LMC.

**Conclusion:**

Results show TxA is prevalent (compromising cancer survival for 1 in 7 children globally), confirm the suspected high burden of TxA in LMC, and illustrate the negative impact of poverty on its occurrence. The present estimates may appear small compared to the global burden of child death from malnutrition and infection (measured in millions). However, absolute numbers suggest the burden of TxA in LMC is nearly equivalent to annually losing all kids diagnosed with cancer in HIC just to TxA, without even considering deaths from disease progression, relapse or toxicity–the main causes of childhood cancer mortality in HIC. Results document the importance of monitoring and addressing TxA as part of childhood cancer outcomes in at-risk settings.

## Introduction

Treatment refusal or abandonment (TxA), defined as the failure to start or complete curative therapy in pediatric cancer, is a rare phenomenon in high-income countries (HIC), usually studied case-by-case for ethical analysis, and sometimes considered criminal.[1–4] In contrast, in low- and middle-income countries (LMC), TxA has been consistently reported as an important contributor to treatment failure and death.[4, 5] However, most studies from LMC regarding TxA are retrospective reviews [3, 5, 6] or single-institution reports.[7–18] Therefore, estimating the global burden of TxA has not been possible due to lack of comparable data.[4, 5] Although each center’s documentation and interpretation of its own TxA rate is most important for understanding and addressing TxA at each center, obtaining and analyzing aggregated data is important to document prevalence (i.e. burden), raise awareness, and promote center-level measurement across at-risk settings.

This study aimed to estimate the frequency and burden of TxA globally and interpret results considering geographical and socioeconomic contexts. Data thoroughly assessing determinants of TxA will be reported separately.

## Materials and Methods

### Strategy

An internet-based survey was conducted to obtain up-to-date information from centers globally. Cure4Kids (www.cure4kids.org) was selected as the website with the broadest representation to achieve such a sample. Cure4Kids is a free online education and collaboration resource dedicated to supporting the care of children with cancer and other catastrophic diseases worldwide.[19] Due to known limitations of survey data including lack of a confirmatory source, response validity and reliability were carefully evaluated.

### Survey

An online, self-administered survey was used (see S1 Text. Survey Tool to review all questions as included in the survey). The survey was evaluated for content validity by members of the International Society of Pediatric Oncology (SIOP) committee on Developing Countries (PODC) Working Group on Treatment Abandonment and piloted for ease of use in a second SIOP PODC Working Group. The survey included close- and open-ended questions, was administered in English, and required about 10–15 minutes for completion.

### Population

Clinicians (including medical oncologists, surgeons, and radiation oncologists), nurses, social workers and psychologists involved in the care of children with cancer were approached. Email addresses were obtained from the Cure4Kids member directory after ethics approval. However, authors never had direct access to the master distribution list. Eligibility was confirmed through two screening questions. Students, data managers, parents and patients were excluded.

### Conducting the survey

Subjects received an individualized email-specific link, four reminders, and details regarding research activity and purpose. The online survey remained open from February 10 to May 10 of 2012. Patient-level data was not collected or analyzed.

### Definitions

#### a) Magnitude of TxA (quantitative query)

Subjects were prompted to a consensus definition,[20] which defined TxA as “failure to initiate” (refusal) or “failure to complete” curative treatment. Then, they were asked to report on the proportion of children newly diagnosed with cancer that abandon therapy at their center by selecting among six discrete categories (0–5%, 6–15%, 16–25%, 26–50%, 51–75% and >75%).

#### b) Likelihood of TxA (qualitative query)

Subjects were asked to report on the likelihood of TxA at their center for 10 individual cancer diagnoses using the scale: “never/almost never”, “rarely”, “sometimes”, “often”, and “always/almost always”. A “don’t know” category was available (see S1 Text. Survey Tool for details).

#### c) Likelihood score

Although the survey captured overall magnitude of TxA using a discrete numeric range, this score helped evaluate intra-rater reliability. The value was obtained from un-weighted summation of responses to the likelihood of TxA for 10 individual diagnoses.

#### d) Economic hardship

Subjects were asked to report on the proportion of indigent families at the center (below the poverty line or with significant financial challenges). The accepted range was 0–100% (free-text) and aggregated in quartiles for reporting. In the absence of patient-level data, the goal was to capture the prevalence of poverty at the center.

### Data Analysis

**Fig 1:**
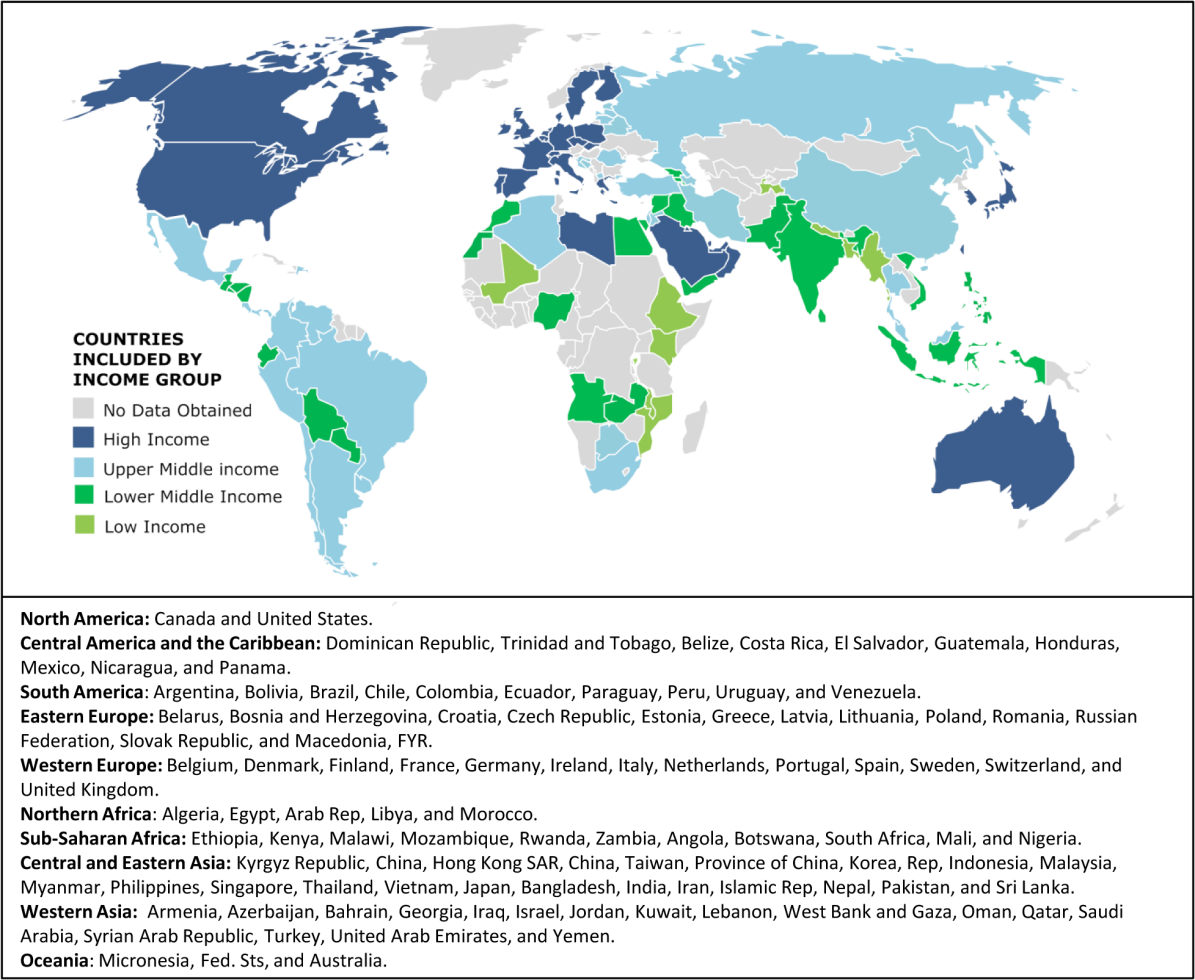
Countries included in the study by World Bank income group classification in 2010 and geographical region. Country names listed are as they appear in World Bank. HIC, high-income countries; UMIC, upper-middle-income countries; LMIC, lower-middle-income countries; LIC, low-income countries. Some countries (such as Chile and Russian Federation) have a higher income group and some countries (such as Libya) have lower income group classification as of 2015, but are illustrated and were kept in the economic bracket assigned through 2010 data in all analyses.

Data was analyzed using Excel and SAS 9.3 and maps were created using SmartDraw and Inkscape. Countries were classified per World Bank Atlas Method[21] by report of gross national income per capita in 2010 into low- (LIC), lower-middle- (LMIC), upper-middle- (UMIC) and high-income- (HIC) country. Of note, some countries presented in Fig 1 (such as Chile and Russian Federation) have a higher income group and some countries (such as Libya) have lower income group classification as of 2015. Because economies and their classifications change over time, for the sake of consistency, all countries were classified based on the 2010 value, regardless of values in previous or later years. A p-value <0.05 was considered significant.

#### a) Estimates

Expected incidence of childhood cancer and magnitude of TxA were calculated by country using the population 0–14 years of age in 2010,[22] the reported cancer incidence for children 0–14 years of age[23], and the mid-point of the median TxA magnitude reported for each country (S1 Table). For countries without incidence data in Parkin et al.[23], the median of reported incidences per million for the income group was calculated and used (HIC 135, UMIC 108, LMIC 101, and LIC 78). Parkin et al. remains the primary source of data on international incidence of childhood cancers, but data is from 1990s and few LMC were included in the volume. As a second source, we used Globocan 2012, which estimates incidence and mortality rates for most countries using data from neighboring countries and other data sources.[24] We queried age-specific cancer incidence rates for each country included and obtained the estimated incidence of “all cancers” for 0–14 year olds. All rates were converted to cases per million. We also queried incidence and mortality cases for “more developed” and “less developed” regions to obtain global survival rates. The only TxA estimate for HIC comes from Germany (0.5%).[25] Therefore, to avoid overestimating the global TxA burden by overestimating TxA in HIC and UMIC, a conservative approach was taken in a second (“adjusted”) set of analyses. In these, TxA magnitude was reassigned to 0.5% for HIC, 1.5% for UMIC, 2.5% for LMIC and 3.5% for LIC. A final set of analyses extrapolated results worldwide (S2 Table).

#### b) Univariable and Multivariable analysis

Binary variables were analyzed with Fisher’s exact test, categorical variables with Chi-square test, and ordinal variables with Mantel-Haenszel Chi-square test. Multivariable analyses were performed for TxA≥6% using logistic regression. Automated forward selection with entry level of 0.1 was used to identify independent predictors.

#### c) Reliability and Validity

Intra-rater reliability was evaluated using Spearman correlation for continuous measures and kappa statistics for specific cut-offs.[26] Spearman correlation (*r*) was considered strong if *r* ≥0.7, moderate if 0.5≤ *r* <0.7, and weak if *r* <0.5. The kappa statistic (*k*) was considered slight if *k* <0.2, fair if 0.21< *k* <0.4, moderate if 0.41< *k* <0.6, substantial if 0.61< *k* <0.8, and almost perfect if *k* >0.81. Validity and inter-rater reliability were assessed by reviewing the following responses: (1) HICs with reported TxA ≥6% and (2) LMC with responses spanning multiple categories, and (3) LMC with single responses that skipped ≥1 category (outliers). Then, if the center information was available, the response was mapped and the respondent’s answers manually reviewed looking for explanations for the chosen TxA category based on geography (capital vs. other), strength of response, and free-text comments. This allowed us to see if the respondent’s choice for the category of TxA was consistent with their report of likelihood of TxA, TxA by phase of treatment, and their free-text comments for phase of treatment, determinants of TxA, strategies to address TxA, and final comments (see S1 Text. Survey Tool to see full survey). As best as possible, we wanted confirmation that in-country (inter-rater) variability was not due to thoughtlessness during the answering process. A response was considered “strong” only if the provider (1) reported estimate came from a database, (2) did not have a database but felt confident about the estimate and other responses were consistent, or (3) provided an explicit explanation. Finally, established socioeconomic indicators have been used as surrogate indicators in pediatric cancer outcomes and TxA research.[27, 28] External validation was pursued using Spearman correlation between reported TxA and established indicators from World Bank[22] and United Nations[29].

### Ethics Statement

Institutional Review Board approval was obtained at St. Jude Children’s Research Hospital and Dana-Farber Cancer Institute.

## Results

### Response rate

The survey was sent to 3,242 email addresses. Of 829 (26%) responses obtained, 729 (88%) subjects met eligibility criteria, 667 (81%) provided demographic information, and 602 (73%) provided completed the sections of interest for this study. There were no major differences between respondents and non-respondents by country, occupation (rate of non-physicians 16% vs. 26%), or preferred language (English for 70% vs. 73%).

### Representativeness

Responses were obtained from 101 countries, including all continents and income groups, but Africa, Oceania and LIC were somewhat under-represented (Fig 1; 36 HIC, 29 UMIC, 26 LMIC, and 10 LIC). We believe that response numbers from Africa, Oceania, and LIC were affected by: 1) internet-based English-language platform, 2) relative scarcity of providers from these contexts eligible to participate (for example, only 14 LIC were represented among respondents and non respondents, therefore, only 55 providers were contacted of which 19 (34%) responded) and 3) low proportion of LIC economies globally (only 34 countries were classified as LIC in 2010). However, ultimately, the 101 countries included in this study represent 85.7% of the world population 0–14 years old or 1.58 billion of 1.85 billion (see S1 Table and S2 Table).

### Respondents

**Fig 2:**
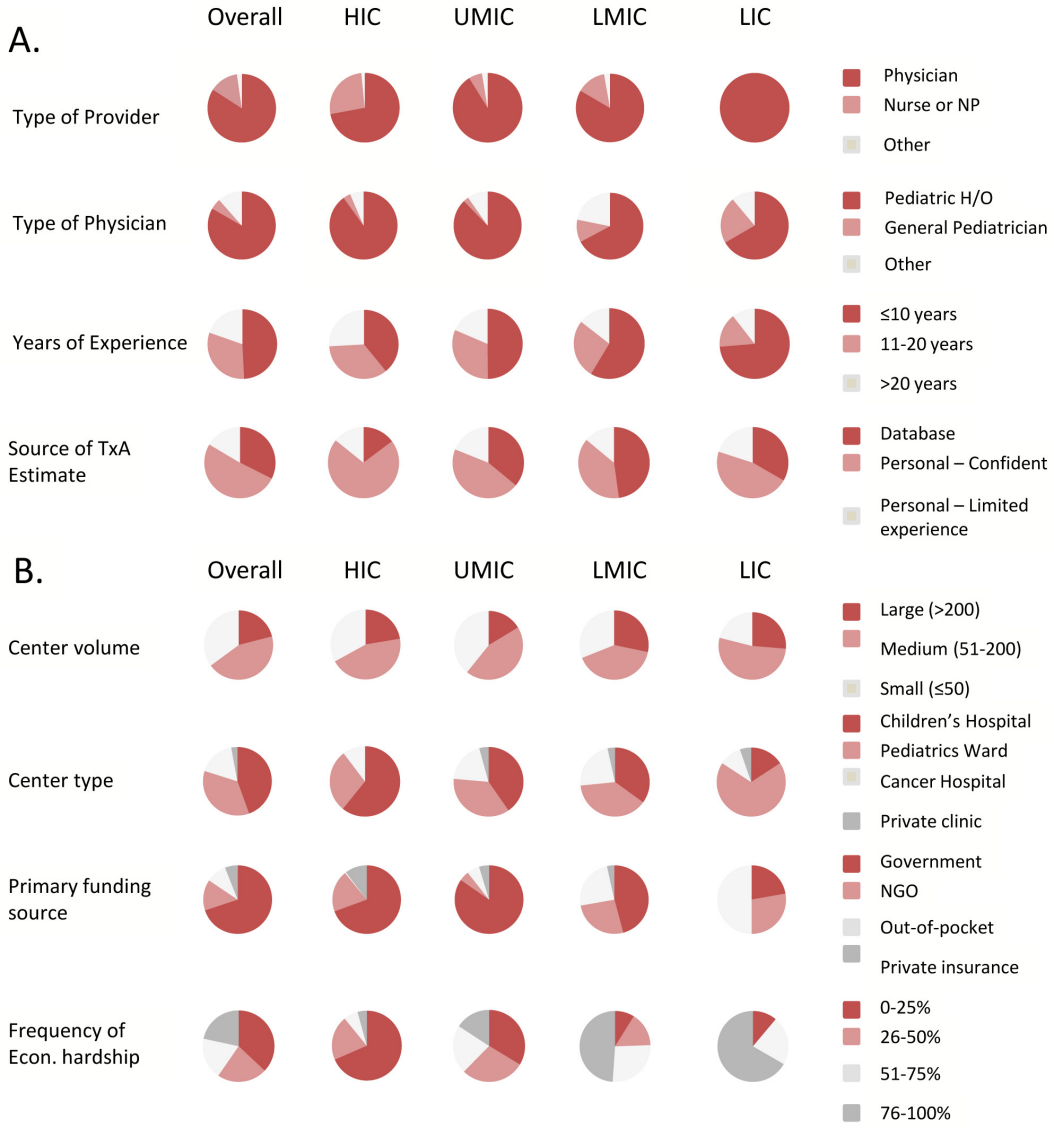
Provider (A) and center (B) demographics. Econ., economic; HIC,high-income countries; H/O, hematology/oncology; LIC, low-income countries; LMIC, lower-middle-income countries; NGO, non-governmental organization; UMIC, upper-middle-income countries. Percentages and further details of other provider and center characteristics are provided in S3 Table.

Subjects were predominantly physicians; pediatric hematologists-oncologists in particular (Fig 2A and S3 Table). There was slight female predominance (58%) overall and higher proportion of physicians with ≤10 years of experience in lower-income countries. Most providers felt confident about their TxA estimate (51%) or reported the estimate came from a database (32%).

### The Centers

Most providers worked in centers seeing >50 new cases per year and only a few providers worked in private clinics (Fig 2B). While government funding was the main source of funding overall, reliance on out-of-pocket expenses was higher in lower income countries. Finally, prevalence of economic hardship <5% was only reported by 4% of subjects. Therefore, even providers in HIC reported a noteworthy frequency of economic hardship among their childhood cancer patients.

### Frequency of TxA by Country

**Fig 3:**
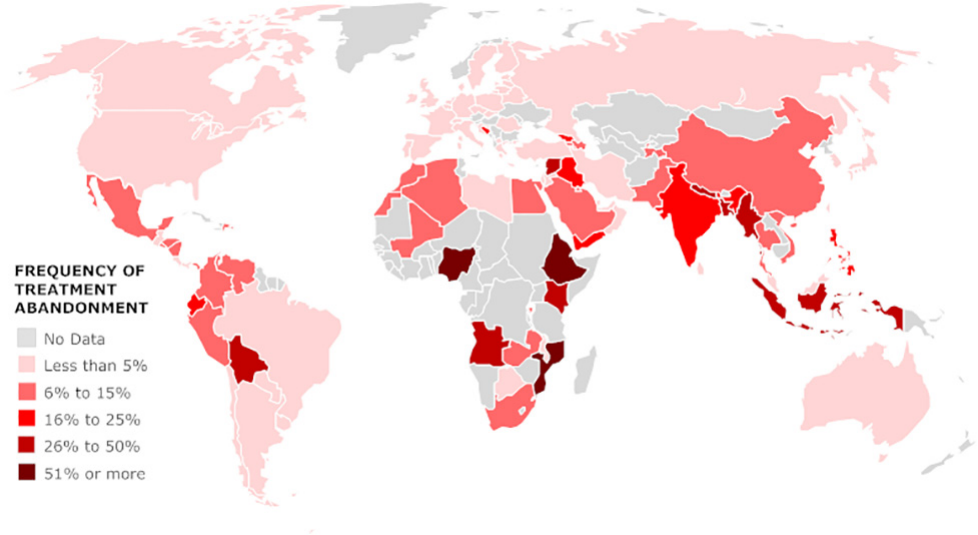
Reported median frequency of treatment abandonment (TxA) by country.

Geographic analysis revealed median TxA≥6% to be prevalent worldwide; except in North America, Europe and Australia (Fig 3). The median frequency of TxA was ≥6% in 44 of 101 countries analyzed: 9% of HIC, 41% of UMIC, 80% of LMIC, and 90% of LIC (p<0.001).

**Fig 4:**
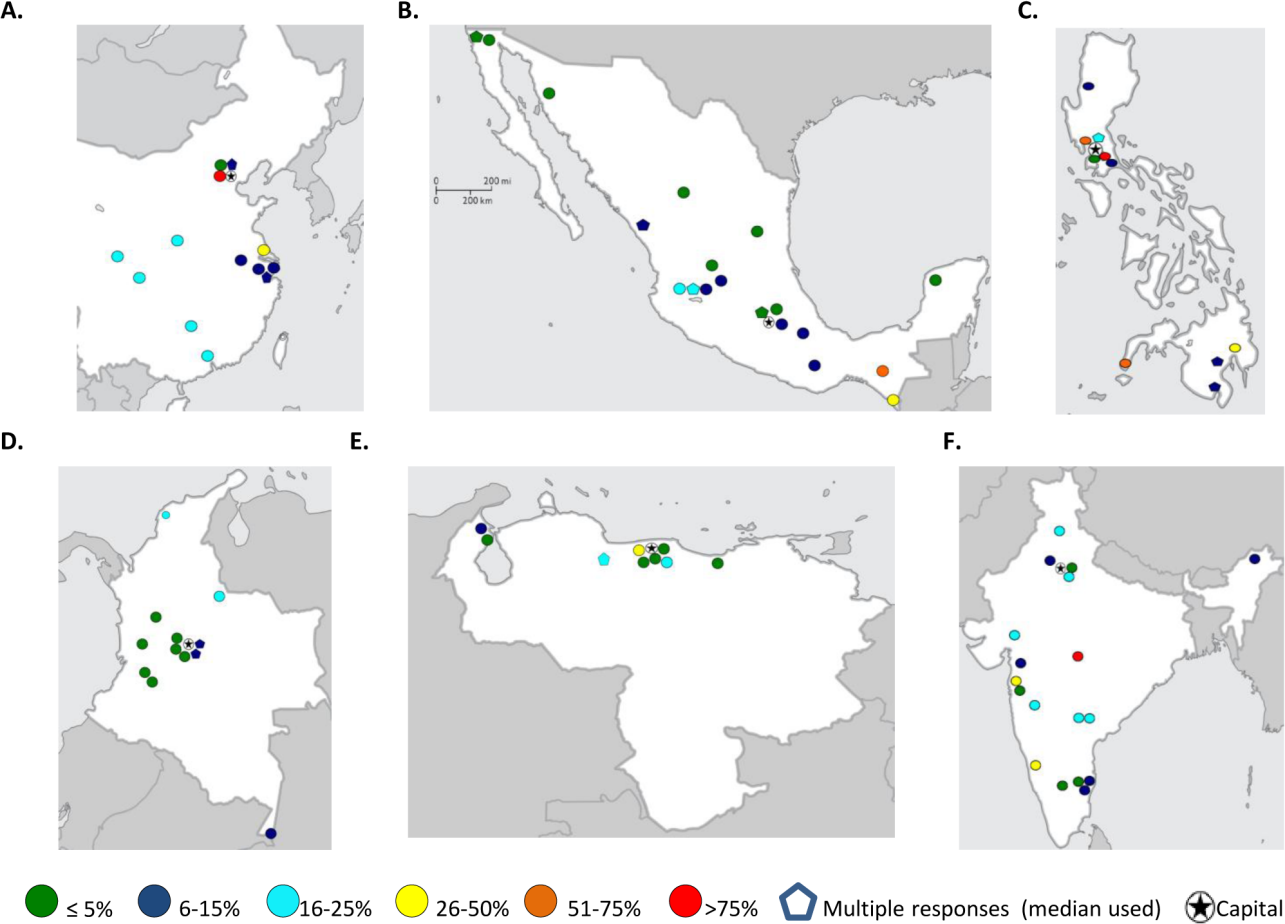
Geographical distribution of treatment abandonment (TxA) rates by city in countries with high in-country variability: China (A), Mexico (B), Philippines (C), Colombia (D), Venezuela (E), and India (F).

Several countries showed significant in-country variability in the rate of TxA. In effort to understand this variability, responses from eleven LMC were manually reviewed (see methods for selection criteria). This manual review by respondent, allowed assessing the respondent’s response to a series of questions in the survey and getting a sense of validity and the respondent’s thoughtfulness in choosing a TxA category (see methods for details). Responses from Philippines, India, China, Mexico, Colombia and Venezuela spanned the widest ranges, and as seen in Fig 4, in some countries, geography appeared to play a role. Responses from Pakistan, Egypt, Morocco, Brazil, and Turkey also met criteria and were manually reviewed. Of 211 responses meeting criteria for manual review, 110 reported TxA≥6%. Of these, most (76%) showed good internal consistency, provided an explicit explanation for the selection or reported having a database as reference for selection. Most (77%) were also from centers outside the capital. Centers reporting ≥75% TxA despite their location in the capital often described primarily serving indigent or referred rural populations. We were not able to identify explicit reasons beyond geography for the observed in-country (inter-rater) variability. Additional factors will be considered during the analysis of other sections of the survey and reported separately.

### Estimated Magnitude of TxA

**Fig 5:**
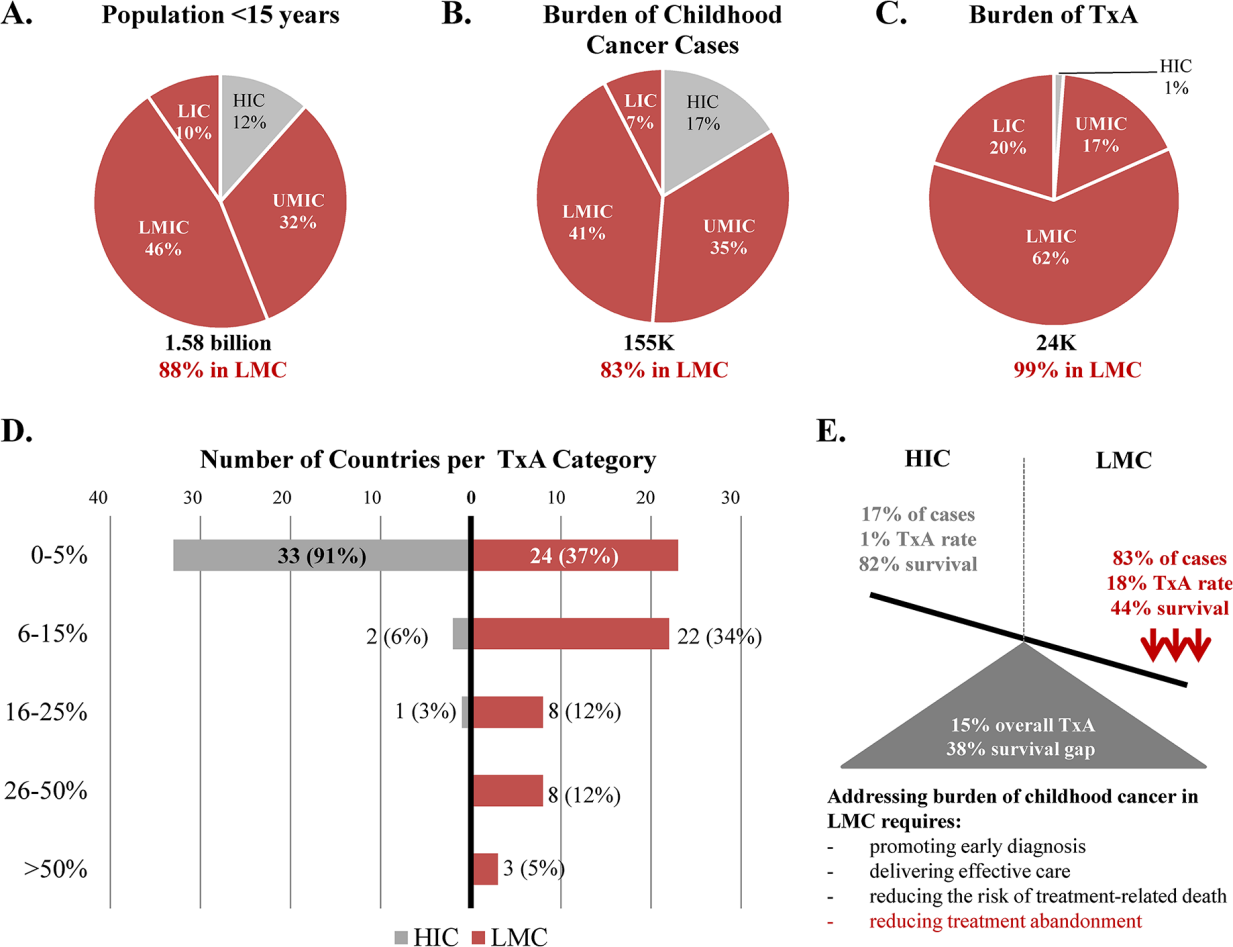
Summary of cancer disparities identified. **Population under 15 years of age (A), burden of childhood cancer cases (B), burden of TxA (C), median reported TxA by country income group (D), and summary of disparities identified (E).** TxA, Treatment abandonment; LMC, Low-and-middle income countries; HIC, high-income countries; UMIC, upper-middle-income countries; LMIC, lower-middle-income countries; LIC, low-income countries.

Based on existing population data and the data collected, 155,088 new cases of cancer were estimated to occur yearly among children 0–14 years of age in the 101 countries analyzed (S1 Table; Fig 5A–5C). Using a conservative (“adjusted”) approach, an estimated 23,854 (15%) of these children abandon therapy. Therefore, 83% of the expected new cases of childhood cancer and 99% of global cases of TxA were attributable to LMC. Using the less conservative (“unadjusted”) approach increased the estimated number of cases of TxA to 24,491 (16%) and reduced the burden attributable to LMC to 97%. Analyses using Globocan 2012 were similar regarding burden of cancer incidence (81%) and TxA attributable to LMC (99% adjusted, 96% unadjusted). Extrapolating results to include all countries worldwide, increased the incidence of childhood cancers to 189,804, the total cases of TxA to 26,298 (14%), and the total cases of TxA in LMC to 26,116 (14%) (S2 Table). However, the burden of cancer incidence and unadjusted TxA attributable to LMC remained similar (86% and 98%, respectively). Therefore, every year TxA compromises cancer survival for about one in seven children globally. Of note, the adjusted estimated worldwide number of children affected by TxA in LMC (26,116) is nearly equivalent to the expected number of childhood cancer cases diagnosed annually in HIC (26,368, S2 Table). The highest burden of TxA was observed in LMIC (Fig 5C), the group of countries with the largest number of children aged 0–14 years (S2 Table).

### TxA and global childhood cancer outcomes

Approximately two thirds of LMC had median TxA≥6% (Fig 5D). Furthermore, by income category, the identified frequency of TxA (15%) splits into 1% in HIC and 18% in LMC (S1 Table). Globocan 2012 estimates suggested childhood cancer survival in more developed regions at 82% and less developed regions at 44% (Table 1). Therefore, TxA explained more than a third of the current survival gap. Fig 5E summarizes the cancer disparities identified.

**Table 1:** Estimated childhood cancer survival by development.

Development Category	Incidence cases (Globocan)	Mortality cases (Globocan)	Mortality Rate	Survival Rate
More developed regions	29,981	5,356	17.9%	82.1%
Less developed regions	133,301	74,600	56.0%	44.0%
Overall	163,282	79,956	49.0%	51.0%

^1^ Incidence and mortality cases for the 0–14 year old age group, including both sexes, were obtained directly from Globocan 2012 for “more developed regions” and “less developed regions”. Available at: http://globocan.iarc.fr/Pages/age-specific_table_sel.aspx. Data retrieved December 29, 2014.

### Univariable and Multivariable Analysis

The evaluation of provider and center characteristics available showed multiple significant associations (Table 2). For example, higher odds of reporting TxA ≥6% was noted for providers practicing in: a) centers located in lower-income countries (compared to HIC), b) centers with large patient volume (>200 new patients per year, compared to centers with <25 new patients per year), c) centers for adult cancer care or general health services (compared to providers practicing in children’s hospitals), d) centers where the primary source of funding was out-of-pocket payments by the family or dependent on non-governmental organizations (NGO), e) centers were >25% of the patients experienced significant economic hardships. Lower odds of reporting of TxA ≥6% was noted among older providers, providers providing an estimate based on their personal opinion (as compared to providers with access to a database), and providers working in centers where the primary source of funding was private insurance. Of these, only the country’s income category, the center’s reliance on out-of-pocket payments as primary source of funding for treatment and, to a lesser extent, higher prevalence of economic hardship, were identified as independent predictors of TxA ≥6% (Table 2). Provider experience was the only provider characteristic independently associated with magnitude of TxA; younger providers reported higher rates of TxA.

**Table 2:** Univariable and multivariable analysis: Predictors of reported TxA ≥6%.

	UNIVARIABLE ANALYSIS				MULTIVARIABLE ANALYSIS		
Predictor	n^1^	Odds Ratio	95% CI	p-value^2^	Odds Ratio	95% CI	p-value^2^
**PROVIDER VARIABLES:**							
Physician (vs. Non-Physician)	602	1.2	0.7, 7.9	0.52			
Years of Experience (ordinal)	602	0.77	0.68, 0.86	<0.001	0.87	0.77, 0.97	0.012
Male Provider	602	1.2	0.85, 1.7	0.31			
Data Source							
Database	195	Ref	Ref	Ref			
Personal opinion, confident	308	0.57	0.39, 0.83	0.003			
Personal opinion, not sure	99	1.4	0.85, 2.2	0.20			
**CENTER VARIABLES:**							
Income Category^3^							
HIC	176	Ref	Ref	Ref	Ref	Ref	Ref
UMIC	275	3.9	2.3, 6.7	<0.001	2.9	1.6, 5.3	0.003
LMIC	136	25.4	13.8, 46.7	<0.001	12.4	5.9, 26.5	<0.001
LIC	15	109.2	13.6, 875.2	<0.001	28.2	3.1–253.3	<0.001
Center Volume^**4**^							
25 and less	88	Ref	Ref	Ref			
26 to 50	122	1.1	0.58, 1.9	0.88			
51 to 100	134	1.3	0.72, 2.3	0.40			
101 to 200	137	1.4	0.82, 2.5	0.21			
More than 200	121	1.9	1.1, 3.4	0.03			
Center Type							
Children’s Hospital	263	Ref	Ref	Ref			
Cancer Hospital	106	1.9	1.2, 3.0	0.009			
General Hospital	211	1.8	1.2, 2.6	0.002			
Private Clinic	17	0.94	0.32, 2.8	0.91			
Primary Funding Source^**4**^							
Government (tax or insurance)	422	Ref	Ref	Ref			
Private insurance	35	0.20	0.06, 0.66	0.008			
Out-of-pocket	59	13.4	6.2, 29.0	<0.0001	5.0	2.1, 11.8	<0.001
National or International NGO	77	1.9	1.1, 3.0	0.015			
Frequency of Economic Hardship^**4**^							
0–25%	206	Ref	Ref	Ref			
26–50%	124	2.5	1.5, 4.2	<0.001	1.01	1.01, 1.02	0.002
51–75%	98	4.8	2.8, 8.1	<0.001			
76–100%	121	10.4	6.1, 17.5	<0.001			

^**1**^ Total sample size is restricted to those who provided a TxA estimate (602); therefore individual samples are smaller than in demographics section (Supplement A3, total sample n = 667).

^**2**^ p = value obtained through likelihood estimates.

^**3**^ HIC = High-income countries, UMIC = upper-middle-income countries, LMIC = lower-middle-income countries, LIC = low-income countries.

^**4**^ Center volume was evaluated as ordinal, source of funding as out-of-pocket vs. others, and economic hardship as continuous variable in multivariable analysis in order to preserve power.

### Data Quality

Provider’s responses showed strong intra-rater reliability between TxA and the likelihood score (*r* = 0.721, p<0.001, *r* = Spearman statistic), but moderate reliability between TxA and economic hardship (*r* = 0.424, p<0.001). Agreement was best between TxA≥6% (which included all responses of TxA 6–15%, 16–25%, 26–50%, 51–75%, and >75%) and a likelihood score >2 (which meant the provider mostly reported TxA to occur either “sometimes”, “often” or “always/almost always” by diagnosis) (*k* = 0.691, *k* = Kappa statistic, 95%CI: 0.631–0.752). At higher cut-offs the intra-rater reliability decreased. This was an expected finding (because the source variables were ordinal rather than interval) and supported use of TxA≥6% as the cutoff for univariable/ multivariable analysis. Furthermore, from a conceptual standpoint, TxA≥6% can be considered clinically significant and using this rather than a higher threshold would allow a more inclusive analysis of associated factors. Intra-rater reliability was best for providers basing responses on a database (*k* = 0.750; 95%CI: 0.656–0.843) compared to those stating personal estimates with confidence (*k* = 0.667; 95%CI: 0.420–0.764) or with limited experience (*k* = 0.592; 95%CI: 0.42–0.764). Although the difference between the groups was not statistically significant (the 95%CI overlaps), the magnitude of *k* for each subgroup supported designation of the first two groups as “strong” responses. Finally, 14 clinicians from six HICs reported maximum TxA ≥6%. Most responses lacked good internal consistency; however, abandonment risk of immigrant patients was one articulated explanation. All indicators explored for external validation correlated significantly with TxA magnitude in the expected direction (S4 Table).

## Discussion

Global data on TxA has been limited; up to now, quantifying the global burden of TxA had not been possible due to scarcity of cancer registries in LMC[27], inconsistent documentation of TxA in published literature,[5] and lack of a consensus definition until 2011.[20] The only meta-analysis to-date included 20 countries and showed only 40% of outcome studies from LMC directly reported TxA rates.[4] In this context, the current study presents a robust addition to the data-gap through its geographic and economic representation as well as internal and external consistency. Furthermore, our study complements other efforts to study TxA locally and internationally as a vital step to address disparities and improve outcomes for children with cancer.

Our results suggest TxA is prevalent and occurs across continents and country-income levels, confirm the suspected high burden of TxA in LMC, and illustrate the negative impact of poverty on its occurrence. We acknowledge the limitations of using survey data and center-level data to obtain country-level estimates. However, estimates analyzed were likely to under- rather than over-estimate the burden of TxA globally and therefore document a notable prevalence and outcome disparity. Furthermore, although the estimates obtained may appear small compared to the global burden of death from malnutrition and infection (measured in millions), the absolute number is nearly equivalent to annually losing all kids diagnosed with cancer in HIC just to TxA (without even considering deaths from disease progression, relapse or toxicity–the main causes of childhood cancer mortality in HIC).

The influence of poverty on TxA was omnipresent in the results. Significant predictors of TxA were almost exclusively socio-economic, suggesting that in addition to the patient-level deprivation reported in single-institution studies, center- and country-level deprivation may also matter. However, young clinicians reported higher TxA rates. Whether this resulted from more knowledge and willingness to report on TxA remains unclear. More details on provider appraisal of TxA determinants will be reported separately, and future studies may help clarify the potential independent value of understanding provider perceptions of center-specific TxA, not only as a reflection of local TxA magnitude, but as a possible factor affecting provider care delivery practices.

Significant in-country variation was noted in some countries’ reported magnitude of TxA, similar to the recent meta-analysis.[4] In our study, in-depth evaluation of these responses supported the potential role of geography. Interestingly, the broadest variability occurred in countries with wide income inequalities such as Philippines, India, Mexico, and China (Gini coefficient 34–47[22]). Therefore, heterogeneity in results appears to reflect in-country disparities and points to the importance of documenting and addressing TxA in all at-risk settings.

Finally, our results highlight the importance of monitoring and addressing TxA in advancing childhood cancer outcomes globally. The global challenge of pediatric cancer is well recognized, but, from a survival standpoint, poorly quantified.[30, 31] Based on the survival gap indentified through Globocan (which totaled 38%) and knowing that the most likely outcome after TxA is death from progressive disease,[15, 32–34] the TxA magnitude of 15% shown in our study suggests that TxA alone may account for at least one third of the survival gap between HIC and LMC. Therefore, in order to address pediatric cancer survival disparities between high- and low-resource settings, cancer-related deaths must be decreased not just by promoting early diagnosis, delivering effective care, and reducing treatment-related death, but also by reducing TxA (Fig 5E). Our demonstration of the reality of TxA globally, particularly for patients living in LMC and impoverished patients in general, supports the need for further awareness and research.

We conclude by addressing methodological limitations of our study. By using an online English-language platform, we may have lowered the chances of collecting information from LIC (10 countries, 19 responses) and some geographical areas (Africa, Oceania). However, when this study was conducted, the Cure4Kids online membership offered the largest and most diverse cohort available to conduct this study. We also acknowledge the limitations inherent to the survey research methodology including the need to rely on standardization, possible recall bias, and the lack of a confirmatory source in particular. Mindful of these methodological limitations, doing this study has nonetheless allowed us to successfully explore the magnitude of treatment abandonment globally, obtain an estimate and document a striking disparity.

## Supporting Information

S1 Table Estimated annual cases of childhood cancer and magnitude of treatment abandonment for countries analyzed. (PDF)

S2 Table Estimated annual cases of childhood cancer and magnitude of treatment abandonment worldwide. (PDF)

S3 Table Provider and center demographics. (PDF)

S4 Table External validation: Correlation between country-level socioeconomic indicators and reported frequency of treatment abandonment. (PDF)

S1 Text Survey Tool. The data presented in this manuscript pertains primarily to questions 1–15 of the survey tool used for data collection. Results for other sections will be summarized in additional manuscripts.
(PDF)
