# Supplementary material for: Magnitude of Treatment Abandonment in Childhood Cancer
Source: PLoS One. 2015 Sep 30;10(9):e0135230. doi: 10.1371/journal.pone.0135230 (PMC4589240; doi:10.1371/journal.pone.0135230)
Supplement: S1 Table — (PDF) [file pone.0135230.s001.pdf]

Table S2: Estimated annual cases of childhood cancer and magnitude of treatment abandonment for countries analyzed[1]

Country[2]	Income Category[3]	Response Rate by Country	Reported Median Magnitude of TxA	Population 0-14 years (as % of population)[4]	Total Population[4]	Calculated population 0-14 years (n)	Incidence of childhood cancer[5] (Parkin)	Incidence of childhood cancer[6] (Globocan)	Expected childhood cancer cases (Parkin)	Expected childhood cancer cases (Globocan)	Expected cases of TxA – adjusted[7] (Parkin)	Expected cases of TxA – adjusted[7] (Globocan)	Expected cases of TxA – un-adjusted[8] (Parkin)	Expected cases of TxA – un-adjusted[8] (Globocan)
Australia	HIC	7	0-5%	14.7%	8,389,771	1,235,380	142	150	176	185	1	1	4	5
Bahrain	HIC	1	0-5%	20.0%	1,261,835	252,798	*135*	119	34	30	0	0	1	1
Belgium	HIC	3	0-5%	16.9%	10,895,785	1,837,739	*135*	158	248	290	1	1	6	7
Canada	HIC	11	0-5%	16.4%	34,126,181	5,603,237	149	162	836	908	4	5	21	23
Croatia	HIC	1	0-5%	15.0%	4,418,000	661,741	169	176	112	116	1	1	3	3
Czech Republic	HIC	1	0-5%	14.0%	10,519,792	1,474,517	125	110	185	162	1	1	5	4
Denmark	HIC	3	0-5%	18.0%	5,547,683	999,162	159	136	159	136	1	1	4	3
Estonia	HIC	2	0-5%	15.3%	1,340,161	205,396	126	103	26	21	0	0	1	1
Finland	HIC	1	0-5%	16.5%	5,363,352	887,042	154	144	136	128	1	1	3	3
France	HIC	1	0-5%	18.4%	65,075,569	11,951,129	136	124	1,623	1,482	8	7	41	37
Germany	HIC	1	0-5%	13.5%	81,776,930	11,021,431	132	179	1,455	1,973	7	10	36	49
Greece	HIC	3	0-5%	14.6%	11,315,508	1,648,519	*135*	105	223	173	1	1	6	4
Hong Kong SAR, China	HIC	2	0-5%	11.5%	7,067,800	813,196	132	132	107	107	1	1	3	3
Ireland	HIC	3	0-5%	21.2%	4,474,356	948,344	*135*	155	128	147	1	1	3	4
Italy	HIC	5	0-5%	14.1%	60,483,385	8,506,005	144	148	1,229	1,259	6	6	31	31
Japan	HIC	3	0-5%	13.4%	127,450,459	17,025,027	125	106	2,126	1,805	11	9	53	45
Korea, Rep.	HIC	3	0-5%	16.4%	49,410,000	8,119,355	108	162	880	1,315	4	7	22	33
Libya	HIC	1	0-5%	30.4%	6,355,112	1,932,964	*135*	96	261	186	1	1	7	5
Netherlands	HIC	2	0-5%	17.7%	16,615,394	2,941,017	135	149	398	438	2	2	10	11
Oman	HIC	1	0-5%	27.2%	2,782,435	755,764	*135*	93	102	70	1	0	3	2
Poland	HIC	4	0-5%	14.8%	38,183,683	5,645,625	113	124	639	700	3	4	16	18
Portugal	HIC	1	0-5%	15.1%	10,637,346	1,607,866	159	201	256	323	1	2	6	8
Qatar	HIC	2	0-5%	13.5%	1,758,793	237,184	*135*	101	32	24	0	0	1	1
Singapore	HIC	6	0-5%	17.4%	5,076,700	883,225	129	140	114	124	1	1	3	3
Slovak Republic	HIC	1	0-5%	15.1%	5,430,099	821,317	129	154	106	126	1	1	3	3
Spain	HIC	25	0-5%	15.0%	46,070,971	6,892,985	143	133	989	917	5	5	25	23
Sweden	HIC	1	0-5%	16.5%	9,378,126	1,550,795	154	138	239	214	1	1	6	5
Switzerland	HIC	1	0-5%	15.2%	7,826,153	1,192,763	144	163	172	194	1	1	4	5
Taiwan, Province of China	HIC	3	0-5%	17.3%	23,126,580	3,695,605	135	.	499	.	2	.	12	.
Trinidad and Tobago	HIC	1	0-5%	20.6%	1,341,465	276,277	*135*	98	37	27	0	0	1	1
United Arab Emirates	HIC	1	0-5%	17.0%	7,511,690	1,279,286	*135*	105	173	134	1	1	4	3
United Kingdom	HIC	7	0-5%	17.4%	62,231,336	10,806,864	124	141	1,335	1,524	7	8	33	38
United States	HIC	54	0-5%	20.1%	309,349,689	62,108,396	149	165	9,236	10,248	46	51	231	256
Kuwait	HIC	1	6-15%	26.7%	2,736,732	730,775	109	141	80	103	8	10	8	10
Saudi Arabia	HIC	12	6-15%	30.3%	27,448,086	8,330,456	*135*	113	1,126	941	113	94	113	94
Israel	HIC	1	16-25%	27.2%	7,623,600	2,076,926	133	146	276	303	55	61	55	61
Argentina	UMIC	17	0-5%	24.9%	40,412,376	10,050,069	*108*	144	1,090	1,447	16	22	27	36
Belarus	UMIC	1	0-5%	15.0%	9,490,000	1,423,424	*108*	151	154	215	2	3	4	5
Botswana	UMIC	1	0-5%	32.6%	2,006,945	653,480	*108*	53	71	35	1	1	2	1
Brazil	UMIC	60	0-5%	25.5%	194,946,470	49,615,363	112	124	5,557	6,152	83	92	139	154
Chile	UMIC	8	0-5%	22.1%	17,113,688	3,785,035	*108*	156	410	590	6	9	10	15

Country[2]	Income Category[3]	Response Rate by Country	Reported Median Magnitude of TxA	Population 0-14 years (as % of population)[4]	Total Population[4]	Calculated population 0-14 years (n)	Incidence of childhood cancer[5] (Parkin)	Incidence of childhood cancer[6] (Globocan)	Expected childhood cancer cases (Parkin)	Expected childhood cancer cases (Globocan)	Expected cases of TxA – adjusted[7] (Parkin)	Expected cases of TxA – adjusted[7] (Globocan)	Expected cases of TxA – un-adjusted[8] (Parkin)	Expected cases of TxA – un-adjusted[8] (Globocan)
Costa Rica	UMIC	1	0-5%	24.9%	4,658,887	1,160,053	136	130	158	151	2	2	4	4
Iran Islamic Rep.	UMIC	9	0-5%	22.9%	73,973,630	16,972,118	*108*	100	1,841	1,697	28	25	46	42
Jordan	UMIC	6	0-5%	37.5%	6,047,000	2,268,285	*108*	109	246	247	4	4	6	6
Latvia	UMIC	1	0-5%	13.8%	2,239,008	310,062	*108*	135	34	42	1	1	1	1
Lebanon	UMIC	5	0-5%	24.8%	4,227,597	1,047,827	*108*	167	114	175	2	3	3	4
Lithuania	UMIC	2	0-5%	14.9%	3,286,820	488,723	*108*	119	53	58	1	1	1	1
Malaysia	UMIC	5	0-5%	30.3%	28,401,017	8,615,952	*108*	103	934	887	14	13	23	22
Panama	UMIC	5	0-5%	29.0%	3,516,820	1,018,372	*108*	74	110	75	2	1	3	2
Romania	UMIC	5	0-5%	15.2%	21,438,001	3,256,850	*108*	137	353	446	5	7	9	11
Russian Federation	UMIC	14	0-5%	15.0%	141,920,000	21,344,192	*108*	132	2,315	2,817	35	42	58	70
Turkey	UMIC	10	0-5%	26.4%	72,752,325	19,180,824	*108*	143	2,080	2,743	31	41	52	69
Uruguay	UMIC	1	0-5%	22.5%	3,356,584	755,682	120	115	91	87	1	1	2	2
Algeria	UMIC	1	6-15%	27.0%	35,468,208	9,593,108	70	141	670	1,353	67	135	67	135
Azerbaijan	UMIC	1	6-15%	20.9%	9,054,332	1,891,469	*108*	93	205	176	21	18	21	18
China	UMIC	25	6-15%	19.5%	1,337,825,000	260,275,487	105	69	27,303	17,959	2,730	1,796	2,730	1,796
Colombia	UMIC	20	6-15%	28.7%	46,294,841	13,304,989	124	97	1,646	1,291	165	129	165	129
Dominican Republic	UMIC	3	6-15%	31.0%	9,927,320	3,080,275	*108*	73	334	225	33	22	33	22
Mexico	UMIC	43	6-15%	29.1%	113,423,047	33,007,194	*108*	114	3,580	3,763	358	376	358	376
Peru	UMIC	6	6-15%	30.0%	29,076,512	8,713,402	104	119	910	1,037	91	104	91	104
South Africa	UMIC	9	6-15%	30.1%	49,991,300	15,062,418	*108*	49	1,634	738	163	74	163	74
Thailand	UMIC	2	6-15%	20.5%	69,122,234	14,194,005	74	111	1,046	1,576	105	158	105	158
Venezuela, RB	UMIC	12	6-15%	29.5%	28,834,000	8,493,238	*108*	67	921	569	92	57	92	57
Bosnia and Herzegovina	UMIC	1	16-25%	15.0%	3,760,149	565,775	*108*	148	61	84	12	17	12	17
Macedonia, FYR	UMIC	1	16-25%	17.6%	2,060,563	362,996	*108*	108	39	39	8	8	8	8
Belize	LMIC	1	0-5%	35.0%	344,700	120,793	*101*	73	12	9	0	0	0	0
Guatemala	LMIC	9	0-5%	41.5%	14,388,929	5,970,440	*101*	59	604	352	15	9	15	9
Micronesia, Fed. Sts.	LMIC	1	0-5%	36.5%	111,064	40,592	*101*	18	4	1	0	0	0	0
Paraguay	LMIC	5	0-5%	33.5%	6,454,548	2,164,544	*101*	75	219	162	5	4	5	4
Sri Lanka	LMIC	1	0-5%	24.9%	20,653,000	5,137,518	*101*	52	519	267	13	7	13	7
West Bank and Gaza	LMIC	1	0-5%	42.1%	3,811,102	1,603,354	101	125	162	200	4	5	4	5
Armenia	LMIC	2	6-15%	20.2%	3,092,072	623,843	*101*	95	63	59	6	6	6	6
Egypt, Arab Rep.	LMIC	9	6-15%	31.5%	81,121,077	25,580,849	101	138	2,594	3,530	259	353	259	353
El Salvador	LMIC	10	6-15%	32.0%	6,192,993	1,980,786	*101*	69	200	137	20	14	20	14
Honduras	LMIC	4	6-15%	36.8%	7,600,524	2,793,890	*101*	60	282	168	28	17	28	17
Morocco	LMIC	6	6-15%	28.0%	31,951,412	8,949,216	*101*	113	905	1,011	90	101	90	101
Nicaragua	LMIC	5	6-15%	34.5%	5,788,163	1,994,793	*101*	52	202	104	20	10	20	10
Pakistan	LMIC	5	6-15%	35.4%	173,593,383	61,398,490	*101*	74	6,207	4,543	621	454	621	454
Vietnam	LMIC	2	6-15%	23.6%	86,927,700	20,515,180	110	69	2,251	1,416	225	142	225	142
Zambia	LMIC	1	6-15%	46.4%	12,926,409	5,994,437	*101*	99	606	593	61	59	61	59
Angola	LMIC	1	26-50%	46.6%	19,081,912	8,886,713	*101*	46	898	409	337	153	337	153
Bolivia	LMIC	6	26-50%	36.1%	9,929,849	3,582,450	*101*	97	362	347	136	130	136	130
Indonesia	LMIC	6	26-50%	27.0%	239,870,937	64,853,427	*101*	107	6,557	6,939	2,459	2,602	2,459	2,602

Country[2]	Income Category[3]	Response Rate by Country	Reported Median Magnitude of TxA	Population 0-14 years (as % of population)[4]	Total Population[4]	Calculated population 0-14 years (n)	Incidence of childhood cancer[5] (Parkin)	Incidence of childhood cancer[6] (Globocan)	Expected childhood cancer cases (Parkin)	Expected childhood cancer cases (Globocan)	Expected cases of TxA – adjusted[7] (Parkin)	Expected cases of TxA – adjusted[7] (Globocan)	Expected cases of TxA – un-adjusted[8] (Parkin)	Expected cases of TxA – un-adjusted[8] (Globocan)
Syrian Arab Republic	LMIC	2	26-50%	36.9%	20,446,609	7,545,669	*101*	110	763	830	286	311	286	311
Nigeria	LMIC	2	51-75%	42.8%	158,423,182	67,828,035	71	49	4,782	3,324	2,989	2,077	2,989	2,077
Ecuador	LMIC	8	16-25%	30.3%	14,464,739	4,387,841	126	140	551	614	110	123	110	123
Georgia	LMIC	2	16-25%	16.6%	4,452,800	737,675	*101*	28	75	21	15	4	15	4
India	LMIC	21	16-25%	30.6%	1,224,614,327	374,586,979	77	63	28,956	23,599	5,791	4,720	5,791	4,720
Iraq	LMIC	2	16-25%	43.2%	32,030,823	13,824,138	*101*	120	1,398	1,659	280	332	280	332
Philippines	LMIC	23	16-25%	35.4%	93,260,798	33,054,074	101	65	3,332	2,149	666	430	666	430
Yemen, Rep.	LMIC	1	16-25%	44.2%	24,052,514	10,638,859	*101*	99	1,076	1,053	215	211	215	211
Malawi	LIC	1	0-5%	45.8%	14,900,841	6,828,308	*78*	210	531	1,434	19	50	13	36
Kyrgyz Republic	LIC	1	6-15%	30.0%	5,447,900	1,636,875	*78*	42	127	69	13	7	13	7
Mali	LIC	1	6-15%	47.2%	15,369,809	7,247,779	78	153	563	1,109	56	111	56	111
Rwanda	LIC	1	6-15%	42.6%	10,624,005	4,530,696	*78*	89	352	403	35	40	35	40
Bangladesh	LIC	3	26-50%	31.3%	148,692,131	46,543,709	*78*	42	3,616	1,955	1,356	733	1,356	733
Kenya	LIC	2	26-50%	42.5%	40,512,682	17,200,407	*78*	127	1,336	2,184	501	819	501	819
Myanmar	LIC	1	26-50%	25.6%	47,963,012	12,297,368	*78*	81	956	996	358	374	358	374
Nepal	LIC	2	26-50%	36.2%	29,959,364	10,841,774	*78*	67	842	726	316	272	316	272
Ethiopia	LIC	1	51-75%	41.5%	82,949,541	34,401,185	*78*	79	2,673	2,718	1,671	1,699	1,671	1,699
Mozambique	LIC	2	51-75%	44.1%	23,390,765	10,312,862	*78*	116	801	1,196	501	748	501	748
**TOTALS (OVERALL)[9]**					**6,160,420,847**	**1,584,082,322**			**155,088**	**139,797**	**23,854**	**20,580**	**24,491**	**21,255**
**% OF TXA (AS % OF TOTAL INCIDENCE)[9]**											**15.4%**	**14.7%**	**15.8%**	**15.2%**
**TOTALS IN LMC[9]**									**129,336**	**112,962**	**23,557**	**20,288**	**23,708**	**20,452**
**LMC BURDEN (AS % OF OVEALL INCIDENCE)[9]**									**83.4%**	**80.8%**	**98.8%**	**98.6%**	**96.8%**	**96.2%**
**TOTALS IN HIC[9]**									**25,752**	**26,835**	**297**	**293**	**783**	**802**
**% OF TXA IN HIC (AS % OF CASES IN HIC)[9]**											**1.2%**	**1.1%**	**3.0%**	**3.0%**
**TOTALS IN LMC[9]**									**129,336**	**112,962**	**23,557**	**20,288**	**23,708**	**20,452**
**% OF TXA IN LMC (AS % OF CASES IN LMC)[9]**											**18.2%**	**18.0%**	**18.3%**	**18.1%**

Notes:
[1] Table is organized first by country income category (see below), then TxA rate (based on categories provided), and finally alphabetically by the country's name.
[2] Country names are shown as they appear in the World Bank Databank. Taiwan Province of China was available in the survey list of countries, but did not have data in either Globocan or World Bank ; population data was obtained from the US Census Bureau Database (https://www.census.gov/population/international/data/idb/informationGateway.php). West Bank and Gaza appear as "State of Palestine" in Globocan.
[3] Country income category: HIC = High-income country, UMIC = Upper-middle-income country, LMIC = lower-middle-income country, LIC= low-income country.
[4] The World Bank: World Development Indicators. Available online at: http://data.worldbank.org/data-catalog/world-development-indicators. Data collected for 2010; retrieved October 15, 2012.
[5] Column shows cases per million. Source:Parkin DM, International Agency for Research on Cancer. International incidence of childhood cancer, Vol. II. Lyon. For countries without primary incidence data in Parkin et al, the median of the reported incidences for the income group was used (therefore, 135 cases/million for HIC, 108 for UMIC, 101 for LMIC, and 78 for LIC). Incidence data for countries without primary data is in *Italics.*
[6] Column shows cases per million. Source: Globocan 2012. Available at: http://globocan.iarc.fr/Pages/age-specific_table_sel.aspx. Data retrieved November 15, 2014. All rates were converted from cases/100,000 to cases per million. Only Taiwan Province of China did not have data in Globocan. West Bank and Gaza appear as "State of Palestine" in Globocan.
[7] Adjustment for income category: To avoid overestimation of TxA in HIC, for countries reporting magnitude of TxA 0-5% range, TxA rate was reassigned to 0.5% if HIC, 1.5% if UMIC, 2.5% if LMIC and 3.5% if LIC.
[8] Un-adjusted calculations used the midpoint of the TxA range.; for example 2.5% for 0-5% and 10% for 6-15%.
[9] Results in red were chosen for presentation in abstract and figures.
