# Supplementary material for: Magnitude of Treatment Abandonment in Childhood Cancer
Source: PLoS One. 2015 Sep 30;10(9):e0135230. doi: 10.1371/journal.pone.0135230 (PMC4589240; doi:10.1371/journal.pone.0135230)
Supplement: S2 Table — (PDF) [file pone.0135230.s002.pdf]

Table S2. Estimated annual cases of childhood cancer and magnitude of treatment abandonment worldwide

Income Category[2]	Reported Median Magnitude of TxA	Adjusted Reported Median of TxA[3]	Midpoint of Reported Median of TxA	Population 0-14 years (as % of population)[4]	Total Population[4] (n)	Population 0-14 years (n)	Incidence of childhood cancer[5]	Expected childhood cancer cases	Expected cases of TxA (adjusted)	Expected cases of TxA (un-adjusted)
High-income countries	0-5%	0.5%	2.5%	0.1731	1,127,916,328	195,315,431	135	26,368	132	659
Upper-middle income	0-5%	1.5%	2.5%	0.2188	2,472,498,041	540,873,991	108	58,414	876	1,460
Lower-middle-income	16-25%	20%	20%	0.3197	2,494,159,560	797,334,693	101	80,531	16,106	16,106
Low-income countries	26-50%	37.5%	37.5%	0.3926	799,803,865	313,984,460	78	24,491	9,184	9,184
**ALL COUNTRIES COMBINED**					**6,894,377,794**	**1,847,508,576**		**189,804**	**26,298**	**27,409**
**% TREATMENT ABANDONMENT (AS % OF TOTAL INCIDENCE)**									**13.9%**	**14.4%**
**ALL COUNTRIES COMBINED (RESTRICTED TO LMC)**								**163,436**	**26,166**	**26,751**
**LMC BURDEN (AS % OF TOTAL BURDEN)**								**86.1%**	**99.5%**	**97.6%**
