# Supplementary material for: Magnitude of Treatment Abandonment in Childhood Cancer
Source: PLoS One. 2015 Sep 30;10(9):e0135230. doi: 10.1371/journal.pone.0135230 (PMC4589240; doi:10.1371/journal.pone.0135230)
Supplement: S3 Table — (PDF) [file pone.0135230.s003.pdf]

Table S3. Provider and center demographics*

	Total		HIC		UMIC		LMIC		LIC	
**Responses**	**667**		**197**		**306**		**145**		**19**	
**Countries**	**101**		**36**		**29**		**26**		**10**	
	**n**	**%**	**n**	**%**	**n**	**%**	**n**	**%**	**n**	**%**
**Occupation**										
Physician	561	84·1%	142	72%	279	91%	121	83%	19	100%
Nurse/nurse practitioner	91	13·6%	52	26%	19	6%	20	14%	0	0%
Social Worker	1	0·1%	1	1%	0	0%	0	0%	0	0%
Psychologist	10	1·5%	0	0%	6	2%	4	3%	0	0%
Other	4	0·6%	2	1%	2	1%	0	0%	0	0%
**Type of Physician (n=556)**										
Pediatric H/O	463	83·3%	127	90%	244	88%	80	67%	12	67%
Adult H/O	15	2·7%	1	1%	5	2%	8	7%	1	6%
General Pediatrician	29	5·2%	5	4%	7	3%	13	11%	4	22%
General Physician	2	0·4%	0	0%	0	0%	2	2%	0	0%
Other	47	8·5%	8	6%	22	8%	16	13%	1	6%
**Experience (years)**										
5 years and less	169	25%	37	19%	79	26%	45	31%	8	42%
6 to 10 years	159	24%	40	20%	74	24%	40	28%	6	32%
11 to 15 years	106	16%	38	19%	44	14%	22	15%	2	11%
16 to 20 years	102	15%	31	16%	53	17%	17	12%	1	5%
More than 20 years	131	20%	51	26%	57	19%	21	14%	2	11%
**Sex**										
Female	384	58%	110	56%	191	62%	75	52%	8	42%
**Source for opinion on TxA (n=602)**										
Database	195	32%	26	15%	99	36%	65	48%	5	33%
Personal opinon, but confident	308	51%	125	71%	124	45%	52	38%	7	47%
Personal opinion, not experienced	99	16%	25	14%	52	19%	19	14%	3	20%
**Center volume (new cases/year)**										
25 and less	100	15%	30	15%	47	15%	21	14%	2	11%
26 to 50	134	20%	35	18%	73	24%	24	17%	2	11%
51 to 100	145	22%	42	21%	74	24%	26	18%	3	16%
101 to 200	148	22%	46	23%	62	20%	33	23%	7	37%
More than 200	140	21%	44	22%	50	16%	41	28%	5	26%
**Center type (n=661)**										
Children's hospital	294	44%	118	61%	123	40%	50	35%	3	16%
Cancer hospital	114	17%	20	10%	59	19%	33	23%	2	11%
General hospital (children's ward)	234	35%	56	29%	110	36%	55	38%	13	68%
Private clinic	19	3%	0	0%	13	4%	5	3%	1	5%
**Primary funding source (n=655)**										
Government	458	70%	134	69%	254	85%	66	46%	4	22%
Private insurance	39	6%	20	10%	14	5%	5	3%	0	0%
Out-of-pocket by family	63	10%	1	1%	18	6%	35	24%	9	50%
Non-governmental organization	95	15%	38	20%	14	5%	38	26%	5	28%
**Secondary funding source (n=611)**										
Government	159	26%	46	27%	68	23%	39	29%	6	43%
Private insurance	126	21%	62	36%	52	18%	12	9%	0	0%
Out-of-pocket by family	96	16%	21	12%	38	13%	32	24%	5	36%
Non-governmental organization	230	38%	42	25%	133	46%	52	39%	3	21%
**Frequency of economic hardship at center (n=597)**										
0-25%	221	37%	113	68%	94	34%	12	9%	2	11%
26-50%	135	23%	34	21%	80	29%	21	16%	0	0%
51-75%	112	19%	11	7%	61	22%	36	27%	4	22%
>75%	129	22%	7	4%	44	16%	66	49%	12	67%

*** Response rate is 667 unless otherwise specified· See methods for details on definition for frequency of economic hardship. HIC = High-income countries, UMIC = upper-middle-income countries, LMIC = lower-middle-income countries, LIC = low-income countries.**
