# Supplementary material for: Magnitude of Treatment Abandonment in Childhood Cancer
Source: PLoS One. 2015 Sep 30;10(9):e0135230. doi: 10.1371/journal.pone.0135230 (PMC4589240; doi:10.1371/journal.pone.0135230)
Supplement: S4 Table — (PDF) [file pone.0135230.s004.pdf]

Table S5. External validation: Correlation between country-level socioeconomic indicators and reported frequency of treatment abandonment

	n[1]	Spearman Coefficient[2]	95% Conf. Interval	p-value
**Center-level indicators (for comparison)**				
Likelihood of TxA	581	**0.721**	0.680, 0.759	<0.0001
Frequency of economic hardship	549	0.424	0.352, 0.490	<0.0001
**Country-level socioeconomic indicators[3]**				
Health expenditure per capita (current US$)	599	**-0.593**	-0.643, -0.539	<0.0001
GDP per capita (current US$)	602	**-0.558**	-0.611,-0.500	<0.0001
Vulnerable employment, total (% of total)	459	**0.548**	0.480, 0.608	<0.0001
Human Development Index (value)	599	**-0.535**	-0.589, -0.474	<0.0001
Income Category: World Bank Atlas Method[4] (LIC, LMIC, UMIC, HIC, ordinal)	602	**-0.522**	-0.578, -0.461	<0.0001
Mortality rate, under-5 (per 1,000 live births)	596	0.497	0.433, 0.555	<0.0001
Poverty gap at $2 a day (PPP) (%)	412	0.473	0.393, 0.544	<0.0001
Prevalence of undernourishment (% of population)	581	0.455	0.388, 0.517	<0.0001
Life expectancy at birth, total (years)	598	-0.437	-0.499, -0.369	<0.0001
Out-of-pocket health expenditure (% of total expenditure on health)	596	0.413	0.343, 0.477	<0.0001
Economically active children, total (% of children ages 7-14)	283	0.387	0.282, 0.481	<0.0001
Out-of-pocket health expenditure (% of private expenditure on health)	596	0.32	0.246, 0.390	<0.0001
Literacy rate, adult total (% of people ages 15 and above)	470	-0.27	-0.352, -0.184	<0.0001

[1] Variable n is due to availability of the specified indicator for the country in the World Bank database.
[2] Correlation was considered strong if r≥0.7, moderate if r≥0.5 but <0.7, and weak if r<0.5. Bold represents r. ≥0.5.
[3] Indicators obtained from World Bank and United Nations Development Program online databases.
[4] LIC = low-income country, LMIC = lower-middle-income country, UMIC = upper-middle-income country, HIC = high-income country
