# Supplementary material for: Magnitude of Treatment Abandonment in Childhood Cancer
Source: PLoS One. 2015 Sep 30;10(9):e0135230. doi: 10.1371/journal.pone.0135230 (PMC4589240; doi:10.1371/journal.pone.0135230)
Supplement: S1 Text — The data presented in this manuscript pertains primarily to questions 1–15 of the survey tool used for data collection. Results for other sections will be summarized in additional manuscripts. (PDF) [file pone.0135230.s005.pdf]

# A Global Survey on Abandonment of Treatment in Children with Cancer -

## Introduction

You are invited to take part in a research survey study on abandonment of treatment in children with cancer. Abandonment of treatment is a major cause of treatment failure in children with cancer in resource-limited countries. While rare in high-income countries, abandonment may still have major impact on families and health providers when it happens. Despite its significance, abandonment is under-reported. We are conducting this web-based survey to study the extent of abandonment in different places, and to identify related factors and strategies.

You are invited to join this study if you are a doctor, nurse, social worker or psychologist involved in the care of children with cancer. The survey will take 10-15 minutes to complete and will ask questions about the setting in which you work, how abandonment may affect patients, and the factors and strategies that may be used in your setting.

This survey is completely voluntary, thus your work or relationship with any study team members or with Cure4Kids will not be affected by whether you take part in this study. We will also respect your privacy and data collected is confidential and your answers will not be linked to any details that could identify who you are in study reports. While your completed surveys would be most helpful, you have the choice to not take part at all, or to stop answering the survey at any time. If you are disconnected, the link will remember where you left off and you will be able to continue answering questions. If you know someone who is not currently a member of Cure4Kids but may be interested in participating, feel free to forward this invitation to them. Because the link is personalized, they need to contact us at childhoodcancer@gmail.com with their request and we will gladly email them a personalized link.

If you have questions concerning the survey please contact Dr. Ramandeep Singh Arora, Co-Chair of The SIOP PODC (Paediatric Oncology in Developing Countries) Abandonment of Treatment Working Group at childhoodcancer@gmail.com.

By completing this web-based survey, you consent to take part in this research study. We know of no harm that taking part in this study could cause you. You will not benefit directly from taking part in this study.

Please click the following link to start the survey.

Thank you,
Paola Friedrich, Geetinder Kaur, Catherine Lam, Raul Ribeiro, Ramandeep Arora
On behalf of the SIOP PODC Abandonment of Treatment Working Group

***1. Are you a clinician, nurse, social worker or psychologist involved in the care of children with cancer?**

- ○ Yes
- ○ No

***2. Are you a STUDENT in one of these roles (clinician, nurse, social worker or psychologist)?**

- Yes
- No

## Demographic Information

### *3. Please select your OCCUPATION

- Physician
- Nurse/nurse practitioner
- Social Worker
- Psychologist
- Other (please specify)

## Demographic Information

### *4. If you are a PHYSICIAN, how would you best describe yourself?

- Paediatric Haematologist and/or Oncologist
- Adult Haematologist and/or Oncologist
- General Paediatrician
- General Physician
- Other (please specify)

## Demographic Information

***5. HOW LONG have you been caring for children with cancer in a professional capacity?**

- 5 years and less
- 6 to 10 years
- 11 to 15 years
- 16 to 20 years
- More than 20 years

***6. What is your sex?**

- Male
- Female

***7. In which COUNTRY do you work?**

**8. In what CENTER do you work (name of the center/institution)?**

***9. Approximately how many children NEWLY DIAGNOSED with cancer (including children with leukemias, lymphomas, solid tumours and brain tumours) are there in your center over ONE YEAR?**

- 25 and less
- 26 to 50
- 51 to 100
- 101 to 200
- More than 200
- Do not know

***10. Which of the following best describes the CENTER where you work?**

- Children's hospital
- Cancer hospital
- General hospital with a children's ward/section
- Private clinic
- Other (please specify)

**11. At your center, approximately WHAT PROPORTION of families of children with cancer are indigent (below the poverty line, or with significant financial challenges for daily needs, as defined in your area)?**

In percentage (%)

***12. What are the SOURCES OF FUNDING for the care of childhood cancer patients in your setting?**

| | Government (tax or national insurance) | Private insurance | Out of pocket payment by patient/family | National non-profit organisation | International non-profit organisation | Do not know |
|---|---|---|---|---|---|---|
| Primary Source | | | | | | |
| Secondary Source | | | | | | |

Other (please specify)

## Magnitude of Abandonment

We would like you now to answer some questions about abandonment of treatment. While this term may mean diffferent things to diffent people, we define ABANDONMENT as "failure to initiate (which has previously been called REFUSAL) or complete curative treatment" in the context of childhood cancer. Abandonment does not include loss to follow up which occurs when treatment is completed and subsequent appointments are missed.

***13. At your center, approximately WHAT PROPORTION of children newly diagnosed with cancer ABANDON treatment (including those who abandon even before treatment is started)?**

- 5% or less
- 6% to 15%
- 16% to 25%
- 26% to 50%
- 51% to 75%
- More than 75%

***14. Where does this estimate come from? We value responses equally, whether they come from a database or from your personal experience.**

- Estimate is my personal opinion and I feel confident in this estimate
- Estimate is my personal opinion, but I don't know much about this subject
- Estimate comes from a database

## Type of Cancer and Abandonment

***15. For each of the following childhood cancers, how LIKELY is treatment abandonment in your setting?**

| | Never/Almost Never | Rarely | Sometimes | Often | Always/Almost Always | Do not know |
|---|---|---|---|---|---|---|
| Acute lymphoblastic leukaemia | ○ | ○ | ○ | ○ | ○ | ○ |
| Acute myeloid leukaemia | ○ | ○ | ○ | ○ | ○ | ○ |
| Hodgkin's lymphoma | ○ | ○ | ○ | ○ | ○ | ○ |
| Non-hodgkin's lymphoma (including Burkitt's lymphoma) | ○ | ○ | ○ | ○ | ○ | ○ |
| Brain tumours - low grade glioma | ○ | ○ | ○ | ○ | ○ | ○ |
| Brain tumours - medulloblastoma | ○ | ○ | ○ | ○ | ○ | ○ |
| Wilm's tumour | ○ | ○ | ○ | ○ | ○ | ○ |
| Retinoblastoma | ○ | ○ | ○ | ○ | ○ | ○ |
| Soft tissue sarcoma | ○ | ○ | ○ | ○ | ○ | ○ |
| Bone sarcoma | ○ | ○ | ○ | ○ | ○ | ○ |

**16. In your center, at what stage of treatment are children with ACUTE LYMPHOBLASTIC LEUKEMIA highly likely to abandon treatment? (select up to 3 options)**

- ☐ Prior to starting treatment
- ☐ During induction or intensification
- ☐ In maintenance
- ☐ If not responding or relapsing on treatment
- ☐ Does not apply as they do not abandon
- ☐ Other (please specify)

**17. In your center, at what stage of treatment are children with WILMS TUMOUR highly likely to abandon treatment? (select up to 3 options)**

- ☐ Prior to starting treatment
- ☐ During chemotherapy given before surgical removal of tumour (if applicable)
- ☐ Prior to surgical removal of tumour
- ☐ After surgical removal of tumour
- ☐ During chemotherapy given after surgical removal of tumour
- ☐ In relation to radiotherapy
- ☐ Not responding or disease progression on treatment
- ☐ Does not apply as they do not abandon
- ☐ Other (please specify)

**18. In your center, at what stage of treatment are children with BONE SARCOMAS highly likely to abandon treatment? (select up to 3 options)**

- ☐ Prior to starting treatment
- ☐ During chemotherapy given before surgery
- ☐ Prior to surgery (amputation)
- ☐ Prior to surgery (limb-sparing)
- ☐ During chemotherapy given after surgery
- ☐ In relation to radiotherapy
- ☐ Not responding or disease progression on treatment
- ☐ Does not apply as they do not abandon
- ☐ Other (please specify)

## Factors Related to Abandonment

ABANDONMENT is defined as "failure to initiate (which has previously been called REFUSAL) or complete curative treatment" in the context of childhood cancer. Abandonment does not include loss to follow up which occurs when treatment is completed and subsequent appointments are missed.

### *19. How are the following FACTORS related to the LIKELIHOOD of abandonment in your setting?

| | Strongly decreases likelihood | Decreases likelihood | No relation | Increases likelihood | Strongly increases likelihood |
|---|---|---|---|---|---|
| Younger child | ◯ | ◯ | ◯ | ◯ | ◯ |
| Older child or adolescent | ◯ | ◯ | ◯ | ◯ | ◯ |
| Female child | ◯ | ◯ | ◯ | ◯ | ◯ |
| Male child | ◯ | ◯ | ◯ | ◯ | ◯ |
| Undernourished child | ◯ | ◯ | ◯ | ◯ | ◯ |
| HIV positive child | ◯ | ◯ | ◯ | ◯ | ◯ |
| Low parental education | ◯ | ◯ | ◯ | ◯ | ◯ |
| Low socioeconomic status | ◯ | ◯ | ◯ | ◯ | ◯ |
| Long travel time to center | ◯ | ◯ | ◯ | ◯ | ◯ |
| Adverse effects and toxicity of treatment | ◯ | ◯ | ◯ | ◯ | ◯ |
| Painful diagnostic and therapeutic procedures | ◯ | ◯ | ◯ | ◯ | ◯ |
| Insufficient communication by health care professionals | ◯ | ◯ | ◯ | ◯ | ◯ |
| Preference of complementary & alternative medicine | ◯ | ◯ | ◯ | ◯ | ◯ |
| Strongly held faith or religious beliefs | ◯ | ◯ | ◯ | ◯ | ◯ |
| Belief in the "incurability" of cancer | ◯ | ◯ | ◯ | ◯ | ◯ |

### 20. Are there ANY OTHER factors related to abandonment in your setting?

1 ____________________

2 ____________________

3 ____________________

## Response to Abandonment

***21. Following diagnosis, if a child with a cancer having a GOOD PROGNOSIS (e.g. standard risk acute lymphoblastic leukaemia or Hodgkin lymphoma) is offered treatment and family/carers refuse to INITIATE it, which of the following would occur in your setting?**

- ○ Their decision would be accepted without discussion
- ○ The family would be counselled but in the end their decision to refuse treatment would be accepted
- ○ The family would be counselled and if needed social services or legal services would be involved
- ○ Does not apply (as it does not occur in our setting)
- ○ Do not know
- ○ Other (please specify)

***22. What about if refusal to INITIATE treatment occurs for a child with cancer having a POOR PROGNOSIS (e.g. metastatic bone or soft tissue sarcomas, high risk neuroblastoma) which of the following would occur in your setting?**

- ○ Their decision would be accepted without discussion
- ○ The family would be counselled but in the end their decision to refuse treatment would be accepted
- ○ The family would be counselled and if needed social services or legal services would be involved
- ○ Does not apply (as it does not occur in our setting)
- ○ Do not know
- ○ Other (please specify)

***23. If the family/carer of the child with a cancer having a GOOD PROGNOSIS (e.g. standard risk acute lymphoblastic leukaemia or Hodgkin lymphoma) who is undergoing treatment, refuses to CONTINUE treatment, which of the following would occur in your setting?**

- Their decision would be accepted without discussion
- The family would be counselled but in the end their decision to refuse treatment would be accepted
- The family would be counselled and if needed social services or legal services would be involved
- Does not apply (as it does not occur in our setting)
- Do not know
- Other (please specify)

***24. What about if refusal to CONTINUE treatment occurs for a child with cancer having a POOR PROGNOSIS (e.g. metastatic bone or soft tissue sarcomas, high risk neuroblastoma) which of the following would occur in your setting?**

- Their decision would be accepted without discussion
- The family would be counselled but in the end their decision to refuse treatment would be accepted
- The family would be counselled and if needed social services or legal services would be involved
- Does not apply (as it does not occur in our setting)
- Do not know
- Other (please specify)

***25. During ongoing treatment, if a child MISSES a SCHEDULED APPOINTMENT for chemotherapy, radiotherapy or surgery, which of the following would occur in your setting in the FIRST INSTANCE?**

- It is not routine practice to contact the child's family/carer
- The child's family/carer would be contacted only if they still do not turn up for the next few days
- The child's family/carer would be contacted on the same/next day
- Does not apply (as it does not occur in our setting)
- Do not know
- Other (please specify)

## Strategies to reduce abandonment

ABANDONMENT is defined as "failure to initiate (which has previously been called REFUSAL) or complete curative treatment" in the context of childhood cancer. Abandonment does not include loss to follow up which occurs when treatment is completed and subsequent appointments are missed.

### *26. How LIKELY are the following strategies to reduce abandonment in your center?

| | Very likely | Moderately likely | Minimally likely | Do not know |
|---|---|---|---|---|
| Locally adapted treatment protocols | ○ | ○ | ○ | ○ |
| Effective procedural sedation and analgesia | ○ | ○ | ○ | ○ |
| Free/subsidised chemotherapy | ○ | ○ | ○ | ○ |
| Free/subsidised supportive care drugs e.g. antibiotics | ○ | ○ | ○ | ○ |
| Free/subsidised blood component therapy | ○ | ○ | ○ | ○ |
| Free/subsidised surgery | ○ | ○ | ○ | ○ |
| Development of a satellite centre | ○ | ○ | ○ | ○ |
| Money for travel | ○ | ○ | ○ | ○ |
| Subsidy for food | ○ | ○ | ○ | ○ |
| Support for lodging e.g. guest house | ○ | ○ | ○ | ○ |
| Patient/parent support group | ○ | ○ | ○ | ○ |
| Detailed and repeated counselling | ○ | ○ | ○ | ○ |
| Patient/Parent information sheets | ○ | ○ | ○ | ○ |

### *27. Of the strategies currently implemented at your center, how HELPFUL have the following strategies been in reducing abandonment in your center?

| | Not available | Very likely | Moderately likely | Minimally likely | Do not know |
|---|---|---|---|---|---|
| Locally adapted treatment protocols | ○ | ○ | ○ | ○ | ○ |
| Effective procedural sedation and analgesia | ○ | ○ | ○ | ○ | ○ |
| Free/subsidised chemotherapy | ○ | ○ | ○ | ○ | ○ |
| Free/subsidised supportive care drugs e.g. antibiotics | ○ | ○ | ○ | ○ | ○ |
| Free/subsidised blood component therapy | ○ | ○ | ○ | ○ | ○ |
| Free/subsidised surgery | ○ | ○ | ○ | ○ | ○ |
| Development of a satellite centre | ○ | ○ | ○ | ○ | ○ |
| Money for travel | ○ | ○ | ○ | ○ | ○ |
| Subsidy for food | ○ | ○ | ○ | ○ | ○ |
| Support for lodging e.g. guest house | ○ | ○ | ○ | ○ | ○ |
| Patient/parent support group | ○ | ○ | ○ | ○ | ○ |
| Detailed and repeated counselling | ○ | ○ | ○ | ○ | ○ |
| Patient/Parent information sheets | ○ | ○ | ○ | ○ | ○ |

**28. Are there ANY OTHER strategies which could be implemented in your setting in the FUTURE to reduce abandonment?**

1

2

3

## Final Questions

**29. Would you like to know the results of the survey?**

- Yes
- No

**30. Would you be interested in taking part in a focus group discussion in the future regarding abandonment of treatment in children with cancer in your setting?**

- Yes
- No

**31. If you said YES to either of the above two questions, kindly provide your email address in the space below**

**32. If you have any further comments about abandonment of treatment in children with cancer or about this survey, kindly use the space below**

Thank you for your time. Your response is very important to us. If you any other comments or would like further information kindly email childhoodcancer@gmail.com
